# Spectral CT: Current Liver Applications

**DOI:** 10.3390/diagnostics13101673

**Published:** 2023-05-09

**Authors:** Ana P. Borges, Célia Antunes, Filipe Caseiro-Alves

**Affiliations:** 1 Medical Imaging Department, Coimbra University Hospitals, 3004-561 Coimbra, Portugal; 2 Faculty of Medicine, University of Coimbra, 3004-504 Coimbra, Portugal; 3 Academic and Clinical Centre of Coimbra, 3000-370 Coimbra, Portugal

**Keywords:** dual-energy CT, spectral CT, liver disease, pancreatic disease, dual-source CT, fast kVp switching, dual-layer detector CT, split-filter, image quality, photon counting

## Abstract

Using two different energy levels, dual-energy computed tomography (DECT) allows for material differentiation, improves image quality and iodine conspicuity, and allows researchers the opportunity to determine iodine contrast and radiation dose reduction. Several commercialized platforms with different acquisition techniques are constantly being improved. Furthermore, DECT clinical applications and advantages are continually being reported in a wide range of diseases. We aimed to review the current applications of and challenges in using DECT in the treatment of liver diseases. The greater contrast provided by low-energy reconstructed images and the capability of iodine quantification have been mostly valuable for lesion detection and characterization, accurate staging, treatment response assessment, and thrombi characterization. Material decomposition techniques allow for the non-invasive quantification of fat/iron deposition and fibrosis. Reduced image quality with larger body sizes, cross-vendor and scanner variability, and long reconstruction time are among the limitations of DECT. Promising techniques for improving image quality with lower radiation dose include the deep learning imaging reconstruction method and novel spectral photon-counting computed tomography.

## 1. Introduction

Spectral or dual-energy computed tomography (DECT) has improved image contrast resolution by means of acquiring data at two different X-ray tube energy levels, allowing for the differentiation and quantification of tissue elements and materials with different attenuation properties at different energy levels, including those displaying similar attenuation at single-energy computed tomography (SECT), such as calcium and iodine [1,2].

Although DECT was first described in the mid-1970s, its introduction into clinical practice was only possible in 2006 due to technological advances [1,2]. There are different DECT platforms available: dual-source systems, consisting of two independent X-ray tubes operating at different energy levels which are then coupled with two independent detectors mounted orthogonally within the rotating gantry; and systems with a single source. The latter group includes systems with a source capable of rapidly switching between two energy levels, those with spectral separation at the level of a specialized detector composed of two layers with different energy sensitivity, and those with a filter that divides the spectrum into high- and low-energy beams at the source [1,2,3]. These systems have variable hardware requirements and diagnostic performances, subject to continuous improvement. Recently, photon-counting detector computed tomography (CT) imaging was introduced into clinical practice. This method is capable of counting and measuring the energy of individual photons, enabling improved material decomposition and image quality [4].

The postprocessing of DECT images offers a myriad of opportunities. These include improved contrast-to-noise ratio (CNR) and iodine conspicuity, both of which are capable of ameliorating diagnostic performance and confidence. Additionally, iodine dose reduction is possible, as is a reduction in radiation dose by the creation of virtual non-contrast (VNC) images [1,2,3]. These and many other advantages of DECT have resulted in the method’s widespread use in various clinical applications that keep evolving. In liver imaging, its use is particularly helpful for lesion detection and characterization, accurate staging and treatment response assessment, non-invasive quantification of fat or iron liver deposition and fibrosis, thrombi characterization, and a number of other applications.

The main objective of this article is to review the current applications and challenges of DECT in liver diseases.

## 2. Materials and Methods

We conducted a comprehensive narrative review of the literature on the most relevant current applications of DECT in liver pathology. We searched MEDLINE and Embase databases for English or Portuguese papers published from January 2010 until December 2022, using the search terms “((((dual energy) OR (spectral)) AND ((computed tomography) OR (CT))) AND ((liver) OR (hepatic))) AND ((((((((((disease) OR (pathology)) OR (diagnosis)) OR (abnormalities)) OR (applications)) OR (advantages)) OR (drawbacks)) OR (limitations)) OR (future)) OR (advances))”. Out of 1358 articles, we excluded 1 retracted and 985 duplicates. Out of the remaining 372 papers, those which were most relevant in relation to the search terms were chosen by means of title and abstract analysis, as well as article content when justified.

Representative images were obtained using a single-source twin-beam DECT scanner at 120 kVp (SOMATOM Go.Top, Siemens Healthineers) in the supine position (spiral pitch factor: 0.3; revolution time: 0.33 s; collimation: 38 × 0.6 mm; reconstruction kernel: Qr40; noise reduction iterative reconstruction algorithm: SAFIRE strength 3). Scanning was performed in a dual-energy mode during the late arterial phase or portal venous phase at a fixed delay of 35 and 70 s after the initiation of IV contrast medium injection, respectively. Patients received 150 mL of an intravenous nonionic contrast medium with an iodine concentration of 300 mg I/mL (Iopromide, Ultravist^®^ 300, Bayer AG, Leverkusen, Germany). This was injected into an antecubital fossa vein at a flow rate of 3 mL/s using a power injector (MEDRAD^®^ Salient, Bayer AG, Leverkusen, Germany). The post-processing of the data was performed using Syngo.via^®^ software (Siemens Healthineers, Erlangen, Germany). Figure editing and schematic representations were performed using Microsoft Corporation software (Paint S, Version 7.0.3).

## 3. Results

### 3.1. Postprocessing Techniques

**Figure 1:**
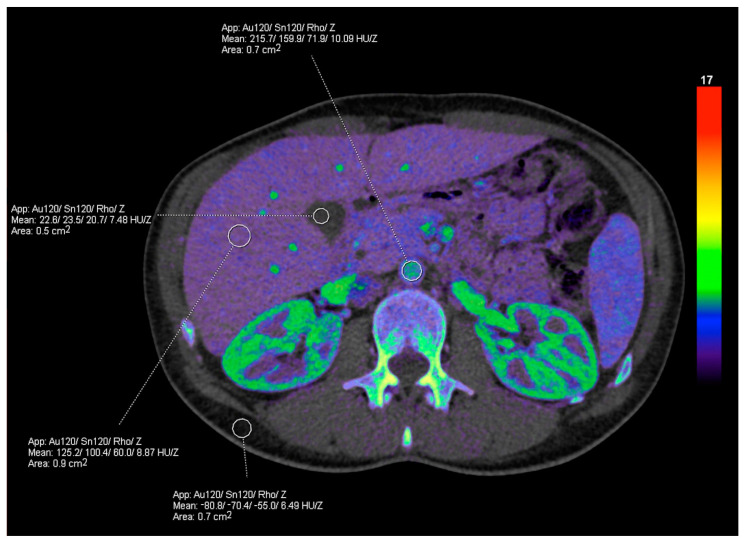
Rho/Z map application. Effective atomic number (Z_eff_) and electron density (Rho) maps allow for the semiquantitative assessment of materials through measurements drawn within a region of interest (ROI), providing Z_eff_ and HU_Rho_ measurements which can be used to calculate the electron density relative to water (ρe). In the Siemens platform, the electron density values (HU_Rho_) are converted into the Hounsfield unit scale, in which water has a value of 0 HU and air has a value of −1000 HU. The effective atomic number (Z) is presented in units of 1. The numbers provided in the measured ROI in this figure refer to the attenuation at the Au120 filter/attenuation at Sn120 filter/Rho value/Z value.

**Figure 2:**
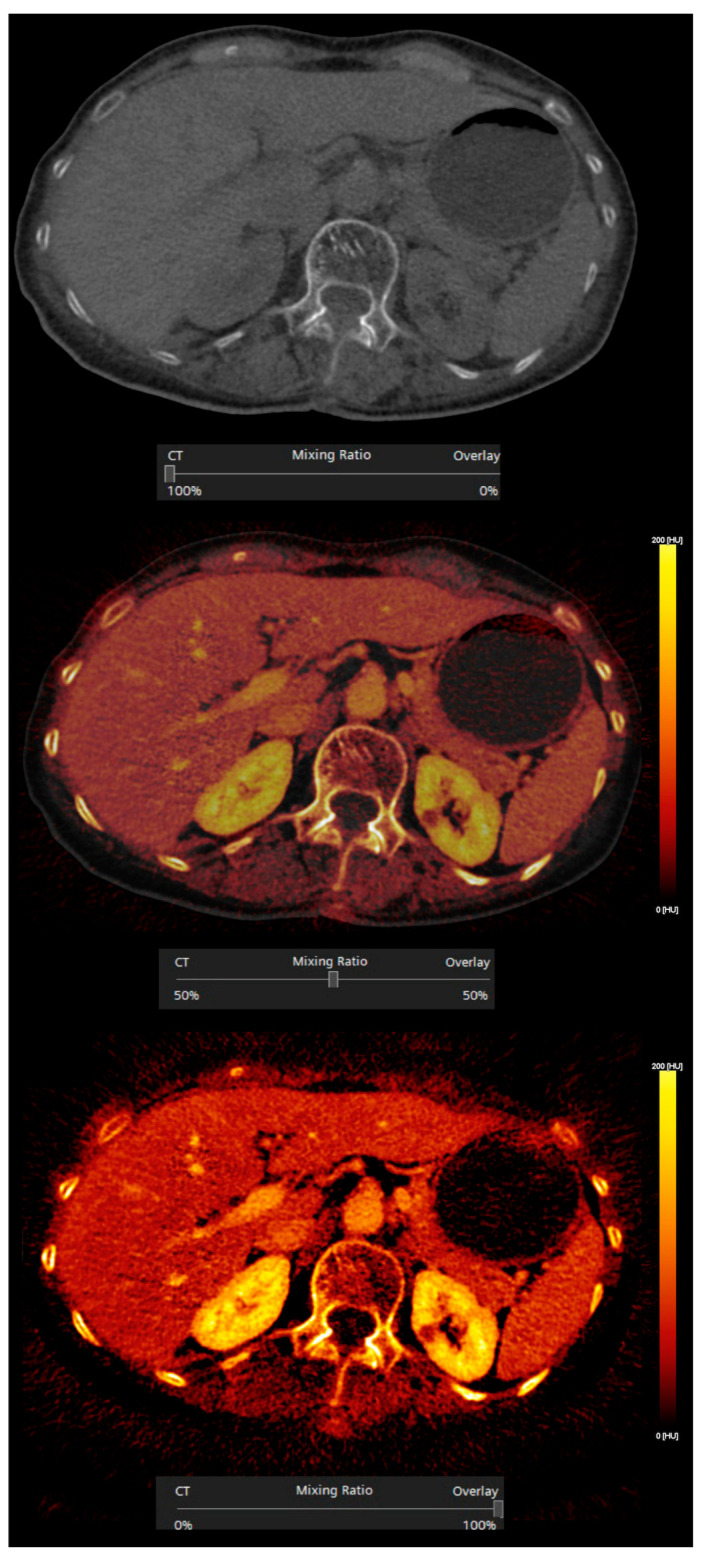
Iodine subtraction and overlay. Iodine may be subtracted, generating virtual non-contrast images (upper image), or superimposed onto grayscale images, generating iodine maps, at a configurable percentage of overlay (50% and 100% in the middle and bottom images, respectively).

The postprocessing of dual-energy data can generate a wide range of useful information by means of material decomposition algorithms, which produce material-selective (decomposition images, mapping atomic number and density) and energy-selective images (reflecting attenuations at a particular photon-energy level). The use of effective atomic number and electron density maps (Figure 1) allow for semiquantitative assessment of materials, providing the calculated concentration of a specified material in units of mass per volume through measurements from a region of interest (ROI). The visual perception of qualitative differences may be improved via color overlay [5]. Different preselected materials (e.g., iodine, fat, calcium) may be quantified, color-coded, or subtracted. For instance, iodine may be superimposed onto grayscale images with a color gradient, generating iodine maps, or it may be subtracted, generating VNC images (Figure 2) [1,6]. The number of “decomposed” materials (usually two or three) depends on each manufacturer’s mathematical model and their density values at different energy levels [7].

Although VNC images have shown reliability in multiple studies and may obviate the need for a true unenhanced acquisition (reducing radiation exposure and scan time), the attenuation values may vary among vendors and with different patient sizes and acquisition phases [2,8,9]. In fact, several studies have shown incomplete iodine subtraction in several abdominal organs, and attenuation differences vary among scanners, software applications, enhancement phase, body tissue, and patient size [10,11]. Additionally, calcifications may appear smaller or be unintentionally subtracted, and pre-existing hyperdense material containing iodine such as lipiodol chemoembolization material may be subtracted and consequently mistaken for contrast enhancement [2,8,12].

**Figure 3:**
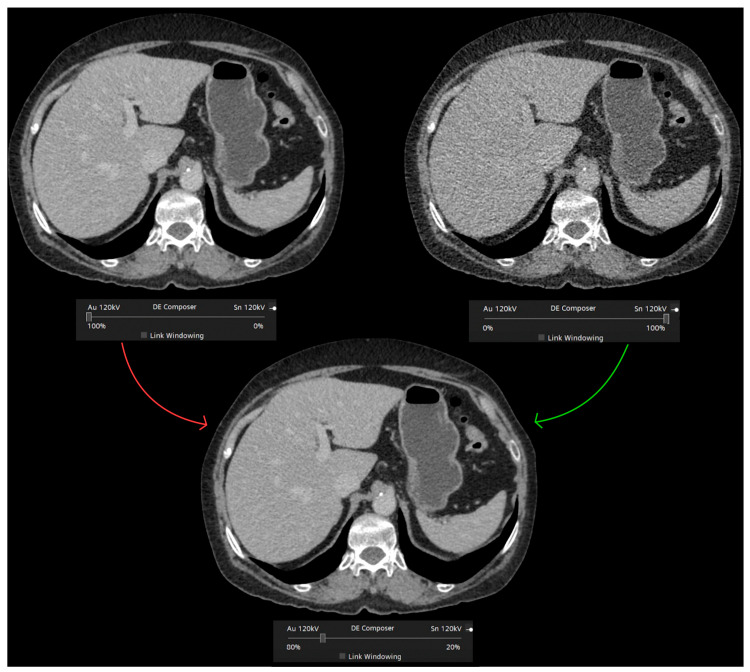
Blended dual-energy image reconstruction. The blended energy values may be chosen by the user, balancing the greater conspicuity of differences in contrast enhancement (at the cost of higher image noise) with low energy values, with the opposite effect of high-energy images. In this example from a TwinBeam DECT scanner (Siemens Healthineers), low- and high-energy beams are derived from a split-filter of gold (Au) and tin (Sn) at the X-ray tube.

Low- and high-energy images may be reconstructed into 120-kilovolt peak (kVp)-like images to simulate the standard SECT scans, but these are less affected by beam-hardening artifacts [1]. Furthermore, the blended energy values may be chosen by the user (Figure 3), balancing the greater conspicuity of differences in contrast to enhancement (at the cost of higher image noise) with low energy values with the opposite effect of high-energy images [7].

**Figure 4:**
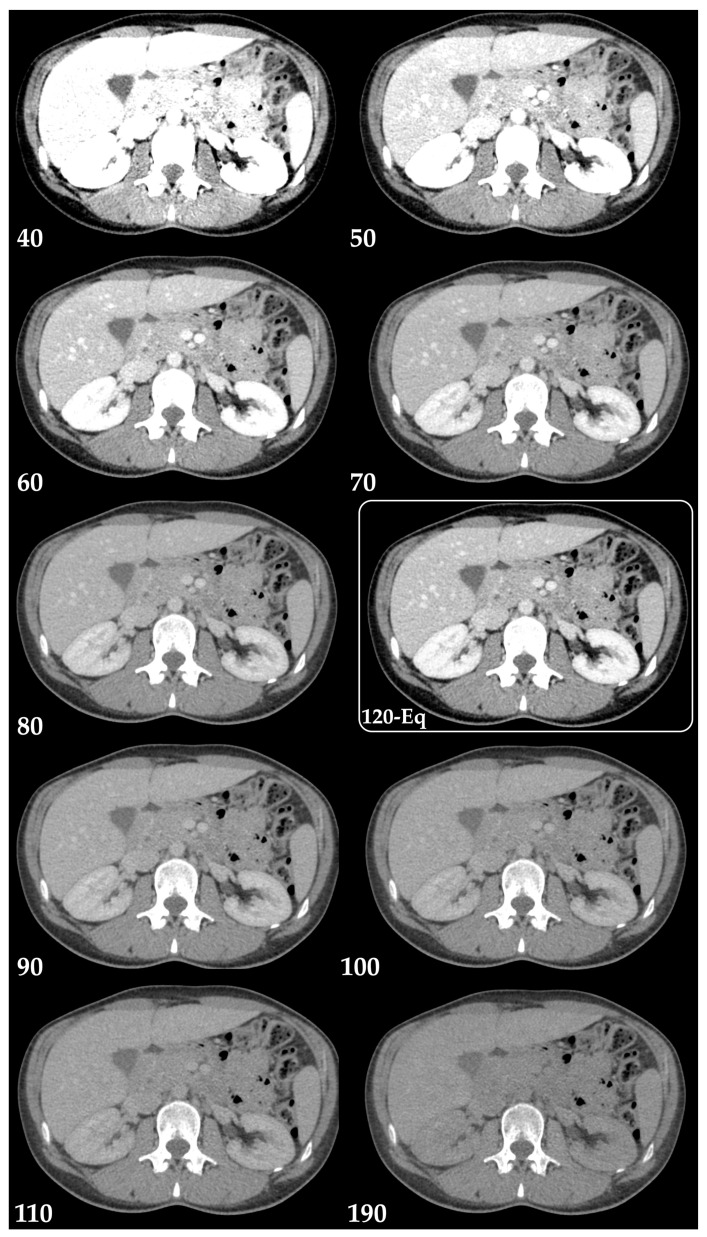
Virtual monochromatic images (Monoenergetic Plus advanced noise-optimized algorithm) from 40 keV to 190 keV in the axial plane (portal venous phase) and mixed 120-kVp-equivalent image (120-Eq). Window settings were kept constant for a more realistic comparability. Note the greater noise with low-keV images and the similarity of the 70 keV VMIs with the blended 120-kVp-equivalent image (120-Eq).

**Figure 5:**
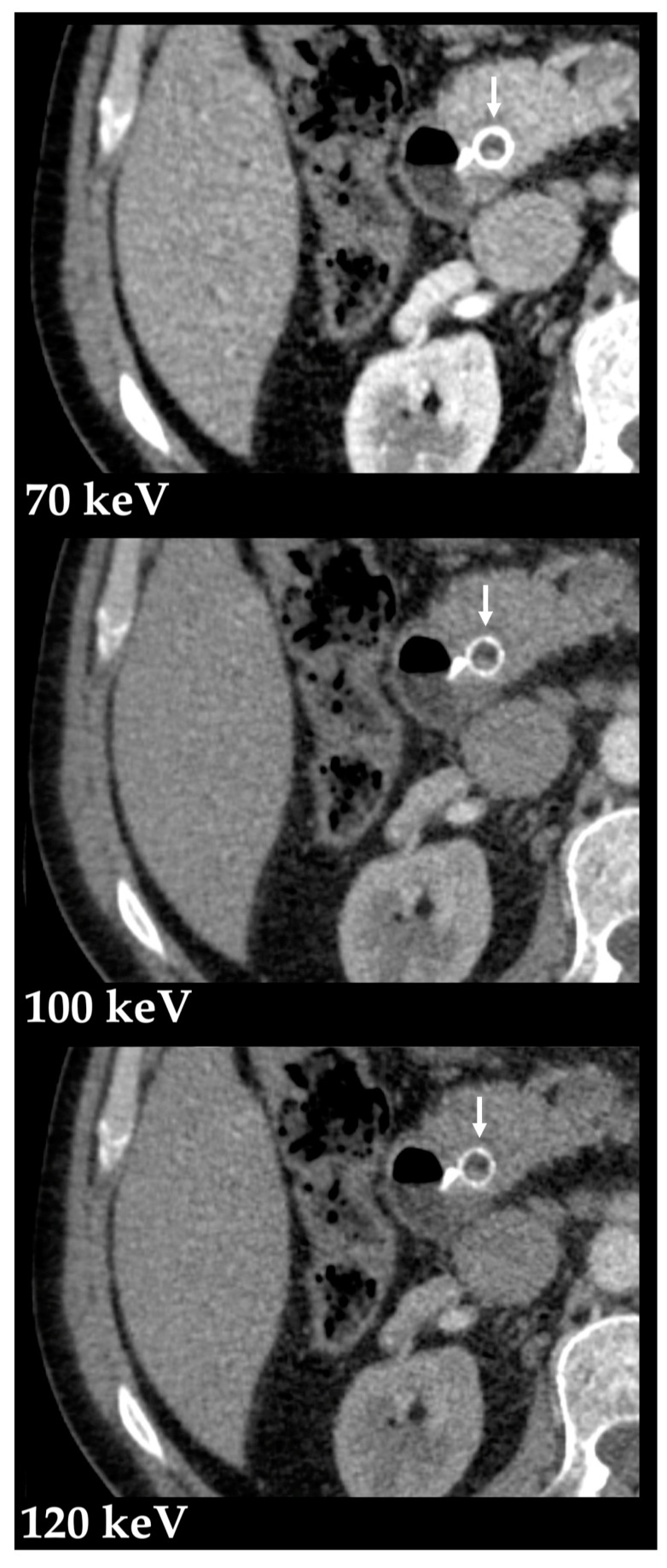
Metal artifact reduction with high-energy VMIs. Virtual monochromatic images (Monoenergetic Plus advanced noise-optimized algorithm) at 70, 100 and 120 keV in the axial plane (portal venous phase) show improved luminal depiction of a metallic biliary stent (arrow) with higher monoenergetic levels due to metal artifact reduction.

Virtual monochromatic images (VMIs) mimic scans obtained at a single energy (Figure 4), described in terms of kiloelectronvolt (keV). Low-keV images improve iodine contrast and lesion conspicuity at the cost of greater noise, whereas high-keV images have less contrast but reduced beam hardening artifacts (Figure 5) and are susceptible to photon-starvation artifacts [6,8]. At 70 keV, these images have shown better objective and subjective image quality compared to conventional polychromatic 120-kVp images at the same radiation dose [13,14].

**Figure 6:**
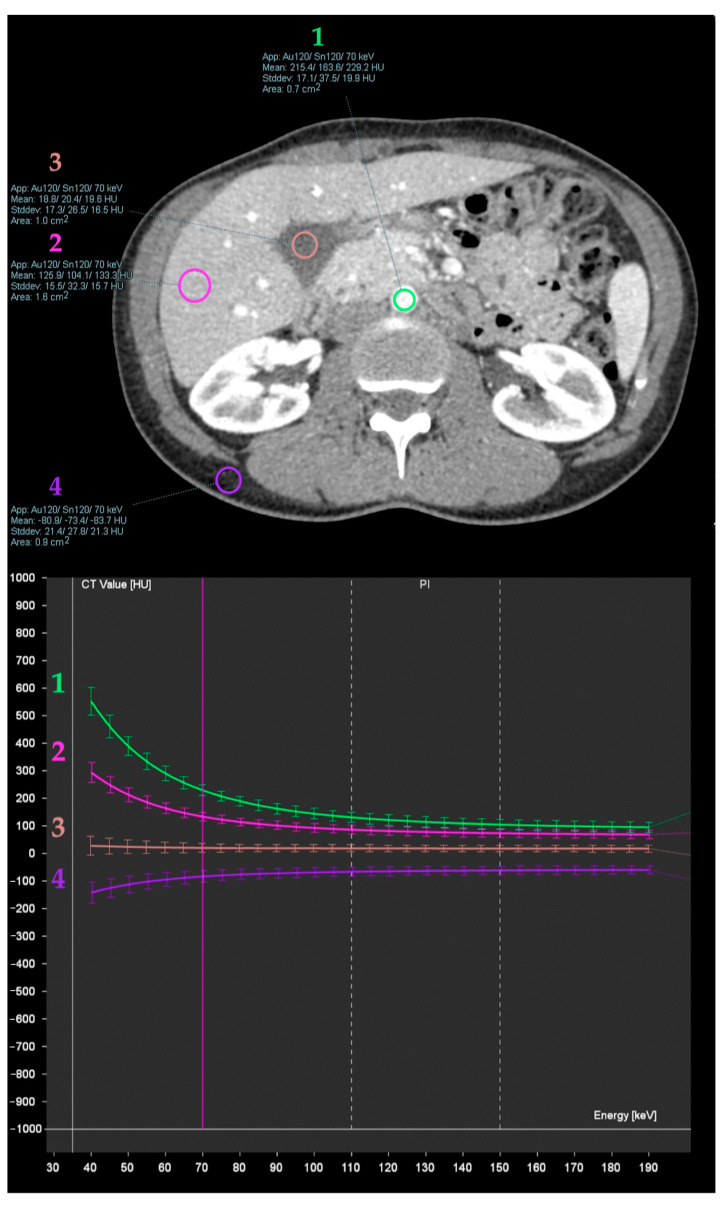
Spectral attenuation curves obtained from iodinated blood in aorta (1), liver parenchyma (2), bile in the gallbladder lumen (3), and abdominal wall fat (4). These are plots of X-ray beam attenuation measurements across a range of monochromatic energy levels, which may be helpful in the characterization of specific materials based on the curve morphology. Note the increasing attenuation of iodine (high atomic number material) at lower energies, as opposed to water materials (stable) and fat (decreasing).

Spectral attenuation curves (Figure 6) are plots of X-ray beam attenuation measurements across a range of monochromatic energy levels (from 40 to 190 keV). These may be helpful in the characterization of specific materials based on the curve morphology [5].

### 3.2. Liver Diseases

#### 3.2.1. Lesion Detection and Characterization

##### Hypervascular Lesions

**Figure 7:**
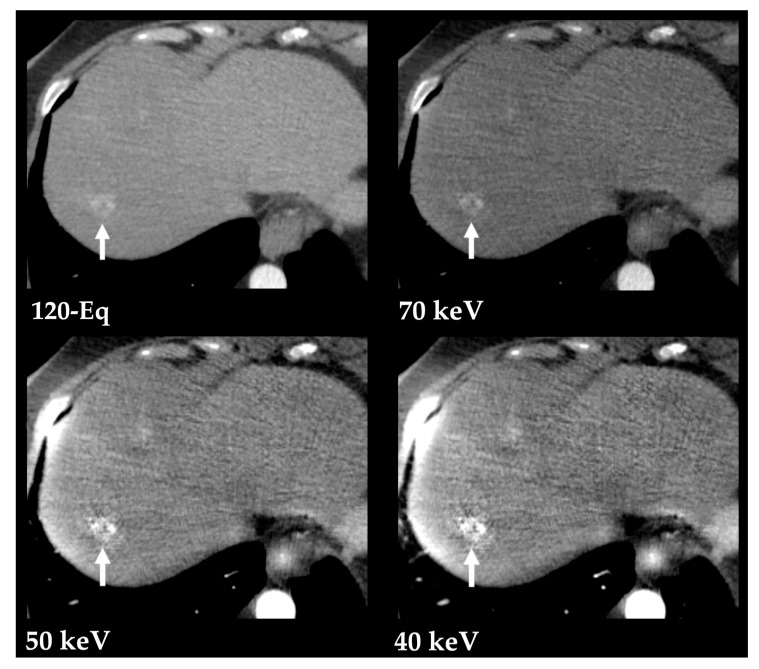
Hypervascular liver metastasis from a pancreatic neuroendocrine tumor, better depicted with low-energy VMIs (70, 50 and 40 keV) obtained from contrast-enhanced DECT in the arterial phase. Postprocessed low-energy VMIs (Monoenergetic Plus advanced noise-optimized algorithm) show the improved conspicuity of the lesion (arrow) compared to the blended 120-kVp-equivalent image (120-Eq) at the cost of increased image noise.

**Figure 8:**
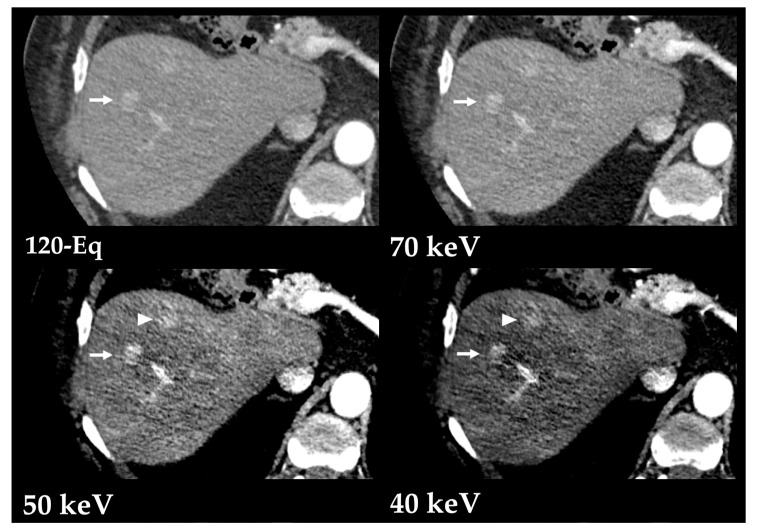
Multifocal hepatocellular carcinoma, better depicted with low-energy VMIs (70, 50 and 40 keV) obtained from contrast-enhanced DECT in the arterial phase. Postprocessed low-energy VMIs (Monoenergetic Plus advanced noise-optimized algorithm) show improved conspicuity of the larger lesion (arrow) compared to the blended 120-kVp-equivalent image (120-Eq) and allow the depiction of a smaller and more subtle lesions at lower keV (arrowhead) at the cost of increased image noise.

Iodine is better depicted at lower-energy states given its low K-edge of 33.2 keV (material-specific minimum energy above which the attenuation peaks) and predominance of photoelectric interaction [2,15]. Therefore, the use of low tube voltage (80 kVp) can improve the visualization of hypervascular focal liver lesions, with lower radiation dose than exhibited by other methods. However, this comes at the cost of greater image noise [15]. Low-keV VMIs derived from DECT (from 40 to 70 keV) improve the detection of hypervascular liver lesions, including hepatocellular carcinoma (HCC) and hypervascular metastases [2,16,17]. Still, the selection of the optimal monoenergetic level is limited by the nonlinear behavior of noise with lowest-energy keV and by the patient body size [3,18]. These limitations were overcome with the development of an advanced noise-optimized VMIs reconstruction algorithm which combines the greater iodine attenuation at low virtual energies with the lower image noise at higher energies, providing improved diagnostic accuracy for detection of hypervascular lesions (Figure 7 and Figure 8) [19]. Optimal monochromatic image sets have been determined in multiple studies (mostly 40 to 50/55 keV, the latter often preferred due to less image noise) [19,20,21,22,23].

Low-energy VMIs also improve the detection of delayed enhancement in tumors with abundant desmoplastic reaction or fibrosis such as cholangiocarcinoma or combined hepatocellular carcinoma–cholangiocarcinoma [24].

Spectral attenuation curves may allow for an accurate discrimination between benign and malignant liver lesions [3,25,26], as well as for the differentiation of primary and metastatic liver neuroendocrine tumors [27].

**Figure 9:**
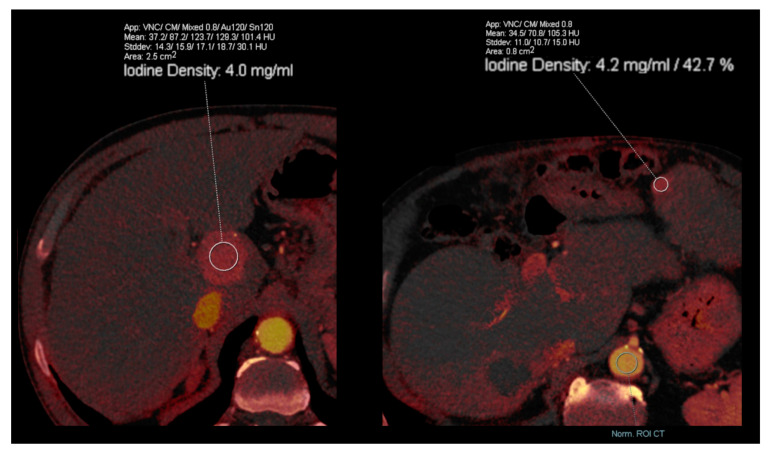
Iodine quantification of hepatocellular carcinomas (HCC). Intratumoral iodine concentration (**left image**) and normalized iodine concentration (**right image**) of HCC in two different patients, measured in the arterial phase. Higher values of preoperative iodine concentrations in the arterial phase predict early recurrence after resection, meaning that they can be valuable predictive biomarkers.

Early recognition of portal vein and microvascular invasion from HCC is crucial for treatment decisions. Conventional CT only depicts larger vessel invasion, but several DECT parameters can accurately predict microvascular invasion [28]. Iodine quantification correlates with microvessel density and is strongly related with perfusion SECT parameters in HCC, with significantly lower radiation dose [29,30]. Combined with perfusion analysis, DECT predicts microvascular invasion, capsular invasion, and tumor grade, although it does so with a higher radiation dose exposure [31]. Peritumoral and intratumoral volumetric iodine concentration (IC), when used during the arterial phase (Figure 9), are useful for predicting microvascular invasion due to the significantly higher peritumoral normalized iodine concentration (NIC) [32]. Higher values of preoperative NIC in the arterial phase predict early recurrence after resection, meaning it can be a valuable predictive biomarker [33].

##### Hypovascular Lesions

**Figure 10:**
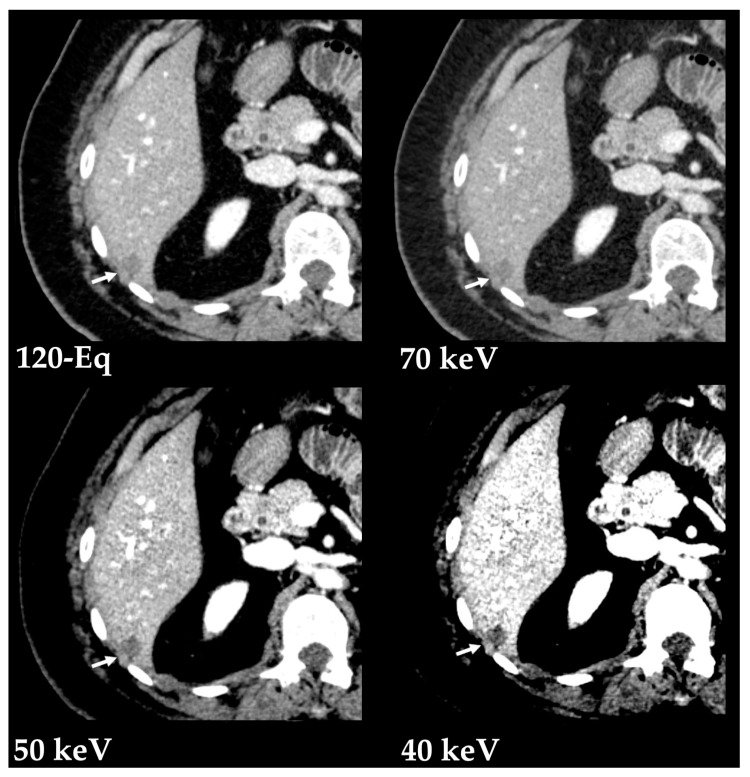
Improved depiction of a hypovascular liver lesion with low-energy VMIs. Contrast-enhanced DECT images in the portal venous phase show a hypovascular liver lesion (arrows). Postprocessed low-energy VMIs (Monoenergetic Plus advanced noise-optimized algorithm by Siemens Healthineers) show improved conspicuity of the lesion margins compared to the blended 120-kVp-equivalent image (120-Eq) at the cost of increased image noise.

The detection of hypovascular liver lesions is also improved with DECT, namely with the use of low kVp data, blending techniques, and monoenergetic imaging [3]. At low-keV images, these lesions show lower attenuation compared to the parenchyma in the portal venous phase (Figure 10) [34]. Optimal reported energy levels for lesion detectability have mostly ranged 40 and 50 keV [35,36,37], but may also be as high s 70 keV [38,39]. A study also reported highest CNR at 190 keV [40]. The improved definition of margins is useful in assessing the extent of diffuse infiltrative lesions [34].

**Figure 11:**
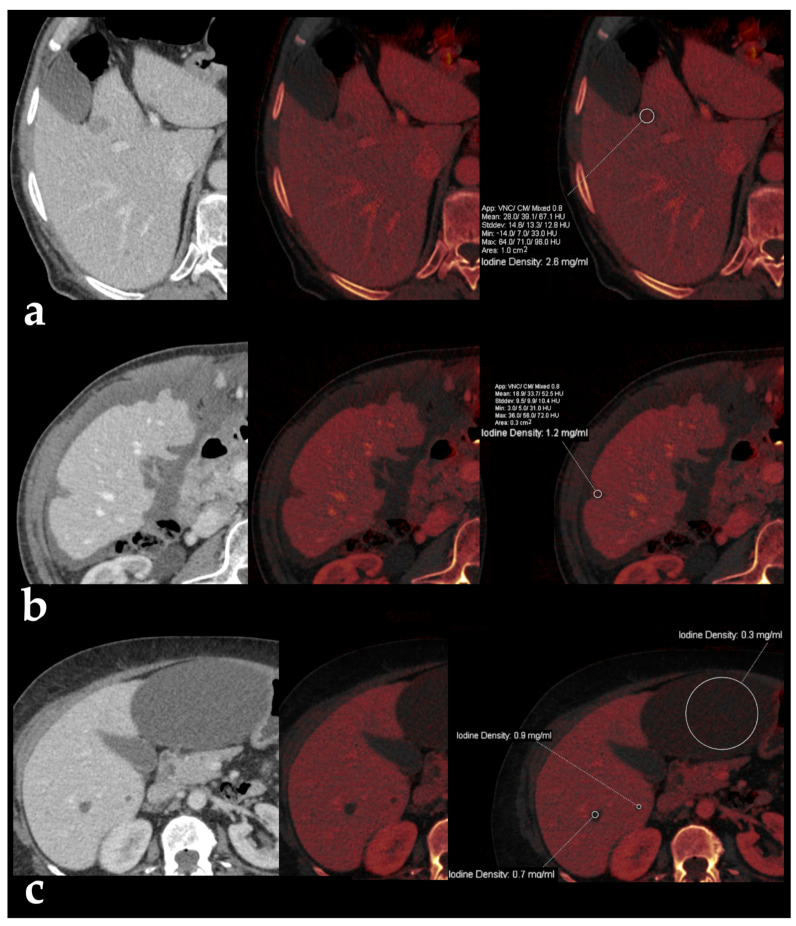
Iodine maps for characterization of hypodense liver lesions. Blended and iodine overlay images from contrast-enhanced DECT images in the portal venous phase in three different patients show small hypodense lesions with iodine densities of 2.6 mg/mL (**a**) and 1.2 mg/mL (**b**) in the cases of liver metastasis from carcinoid tumor and colorectal cancer, respectively, as opposed to simple liver cysts in the bottom images (**c**), which present less than 1 mg/mL of iodine concentration.

**Figure 12:**
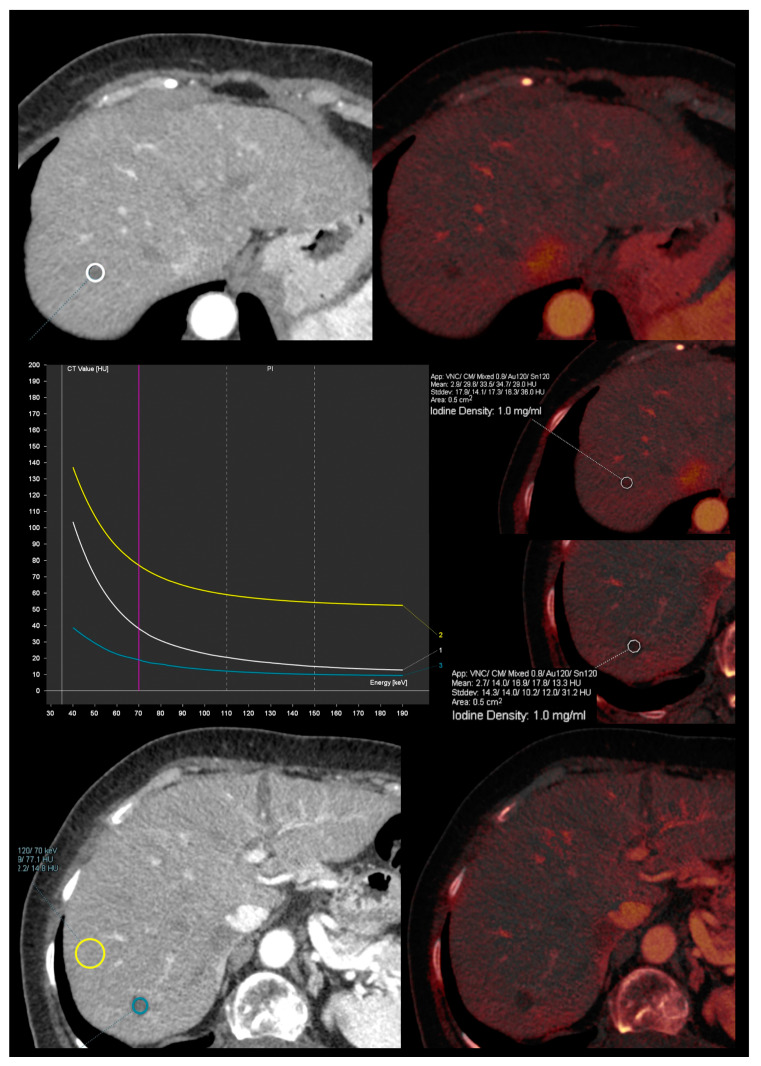
Spectral attenuation curves to confirm the presence of enhancement. Iodine overlay images from contrast-enhanced DECT images in the arterial phase show two small hypodense lesions with iodine density of 1 mg/mL. The superior lesion shows an exponential increase in attenuation at lower energy levels (white curve and circle), as opposed to pseudoenhancement from a cystic lesion, which exhibits a flatter curve (blue curve and circle). The yellow curve and circle correspond to liver parenchyma measurement and is shown for reference.

Iodine maps are helpful in characterizing small hypoattenuating liver lesions, either found incidentally or in patients with a primary malignancy, thus allowing for the distinction between cysts and metastasis based on the absence or presence of iodine within the lesion (Figure 11) [41]. A threshold of 1.2 mg/mL in IC during the portal venous phase (at a fixed delay of 70 s after administration of 150 mL of iopamidol with a concentration of 300 mg I/mL at a flow rate of 3 mL/s) has been found to allow for better differentiation between benign and malignant small hypoattenuating lesions compared to the results obtained from conventional attenuation measurements [42]. In addition, the analysis of the spectral attenuation curve helps to confirm the presence of enhancement in equivocal cases, revealing an exponential increase in attenuation at lower-energy levels, as opposed to pseudoenhancement, which exhibits a flatter curve (Figure 12) [5]. Complex cysts may be also distinguished from simple cysts by means of subtle enhancement depiction [43].

Liver abscesses can be difficult to differentiate from liver metastasis that develops central necrosis or cystic changes. Quantitative analysis performed by DECT has proven to be helpful as metastasis exhibits higher density, effective atomic number and iodine concentration, and lower fat concentration compared to abscesses. Additionally, the IC in the lesion wall in the venous and delayed phases is significantly higher for abscesses [44]. It is also helpful for differentiating liver abscesses from necrotic HCC [45] or small intrahepatic mass-forming cholangiocarcinomas [46].

#### 3.2.2. Treatment Response Evaluation

Sized-based tumor classification systems used to evaluate treatment response, such as version 1.1 of the response evaluation criteria in solid tumors (RECIST 1.1), are limited by variability in lesion target selection or measurement and by the non-consideration of lesion perfusion or composition (lesions with necrosis or myxoid degeneration may remain stable or increase in size despite successful treatment response) [5,6]. This is particularly problematic with emerging immunotherapy agents associated with unconventional response patterns (i.e., pseudo-progression) [6]. Antiangiogenic drugs can also be used to reduce vascularity without changing tumor size. Given its improved capability of vascularity assessment, DECT may serve as a reliable biomarker for tumor viability [5]. The evaluation of HCC angiogenesis with iodine quantification from DECT has shown promising results in animal models [6,47]. Volumetric iodine uptake changes from DECT iodine maps are also valuable for therapy response assessment in advanced HCC patients treated with sorafenib [48].

When evaluating HCC submitted to radiofrequency ablation (RFA), IC allows for accurate differentiation between residual or recurrent HCC, inflammatory reaction zone, and RFA lesions [49]. These parameters have shown the ability to predict the value of HCC progression well within 12 months [50].

In the early assessment after microwave ablation of HCC, 50 keV VMIs provide improved image quality and diagnostic confidence at determining technique efficacy. Additionally, IC is significantly higher in residual tumor compared to the reactive hyperemic rim [51,52].

**Figure 13:**
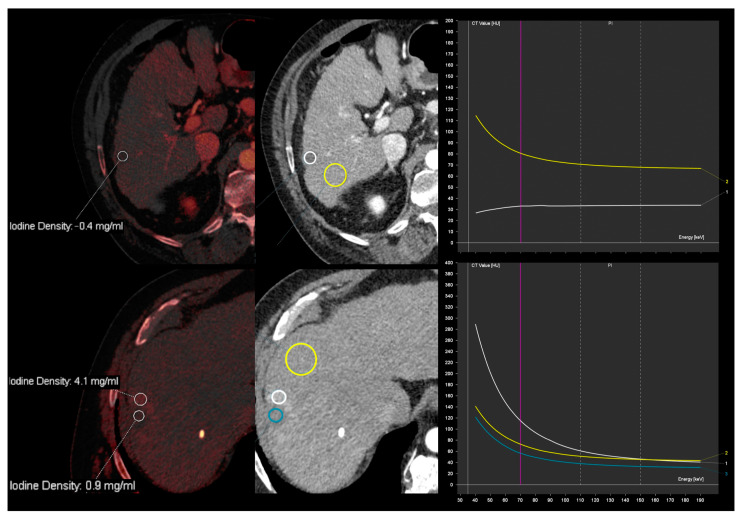
Iodine quantification and spectral attenuation curve analysis obtained from DECT during the arterial phase after transarterial chemoembolization of hepatocellular carcinoma with doxorubicin-eluting microspheres in two different patients. Top images show a hypodense area with iodine density of −0.4 mg/mL and a flat spectral attenuation curve (white curve and circle), consistent with necrotic area. The bottom images show a hypodense area with iodine density of 0.9 mg/mL and a flat spectral attenuation curve (blue curve and circle), with a peripheral thick rim exhibiting iodine density of 4.1 mg/mL and a steep descending spectral attenuation curve (white curve and circle), indicative of active tumor. Yellow curves and circles correspond to liver parenchyma measurements, for reference.

**Figure 14:**
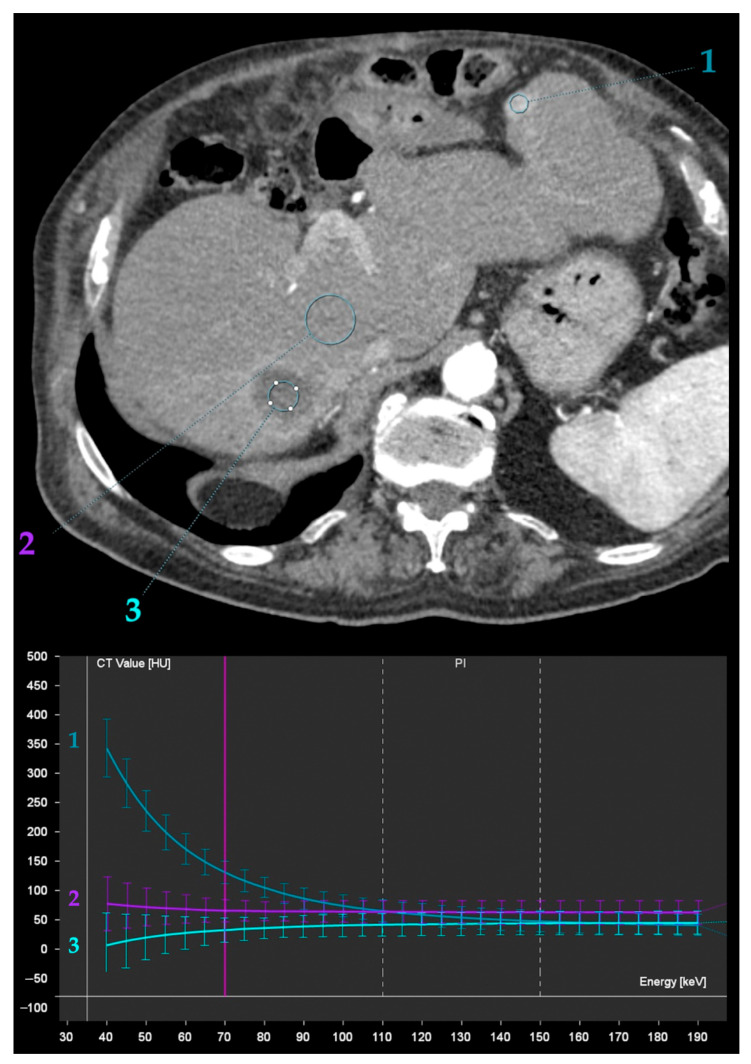
Spectral attenuation curve analysis obtained from DECT during late arterial phase after transarterial chemoembolization (TACE) of hepatocellular carcinoma (HCC) with doxorubicin-eluting microspheres. Recurrent HCC in the left lobe (1) exhibits a steep descending spectral attenuation curve, as opposed to the liver parenchyma (2) and the necrotic area of previous TACE (3).

Transarterial chemoembolization (TACE) is usually performed with a mixture of chemotherapeutic agents and lipiodol, followed by the administration of an embolic agent. Lipiodol deposition within the tumor correlates well with necrosis and predicts tumor recurrence and survival rate. It is not well observed in magnetic resonance imaging (MRI) [53], and its density impairs the detection of enhancing tumor on SECT [54]. Dual-energy CT is capable of lipiodol quantification, allowing more accurate detection of residual or recurrent disease [53,54,55]. Before TACE, NIC values in the arterial phase closely correlates with the grade of lipiodol accumulation in tumors, meaning it may be a parameter used for the selection of patients who are more likely to benefit from the treatment [56]. After TACE, the arterial phase spectral curve is steeper in the active tumor areas compared to necrotic areas, and the arterial iodine fraction is very good for differentiating tumor active area and adjacent normal hepatic parenchyma (Figure 13 and Figure 14) [57]. Iodine overlay images are capable of discriminating between enhanced viable lesions and iodized oil accumulations [58].

A novel embolic bead has been proposed which would use bismuth as the radiopacifier, distinguishable from iodine on DECT, allowing for the identification of non-targeted delivery or undertreated tumors despite the presence of iodinated contrast agent. Furthermore, iodine-based and bismuth-based beads have the capacity to be loaded with different chemotherapy or immunotherapy drugs, allowing dual drug delivery. In vivo safety studies are still needed [59].

The degree of enhancement uptake after yttrium-90 radioembolization has proven to be a valuable tumor response marker [60]. The volumetric iodine uptake changes in intermediate-advanced HCC can predict response and correlate with overall survival [61].

An excellent correlation has also been demonstrated between DECT parameters and the Choi criteria for treatment response evaluation in liver metastasis from gastrointestinal stromal tumor [62]. This task is particularly difficult due to the presence of treatment-related changes including hemorrhage and calcification, which may have less influence on iodine concentration. A recent study introduced DECT vital iodine tumor burden criteria for posttreatment evaluation in these patients; these outperform RECIST 1.1 and mChoi criteria [63].

#### 3.2.3. Diffuse Liver Diseases

Although biopsy is still the reference standard for diagnosing diffuse liver disease, it is invasive and not adequate for monitoring its progression. Regarding non-invasive techniques, MRI is still the most accurate but is contraindicated in some patients [6], expensive and not widely available [64,65].

##### Fat Deposition

Liver steatosis is associated with metabolic syndrome and non-alcoholic fatty liver disease (NAFLD), which affects 30% of the American and European population [66]. It may progress to steatohepatitis and is currently the main reason for cirrhosis development [6,8]. This occurs in about 20% of patients with NAFLD, making early detection of liver fat and implementation of control measurements essential [6]. Chemotherapy also contributes to the increasing incidence of liver steatosis [67]. It may induce a specific form of steatohepatitis and worsens patient prognosis [68].

**Figure 15:**
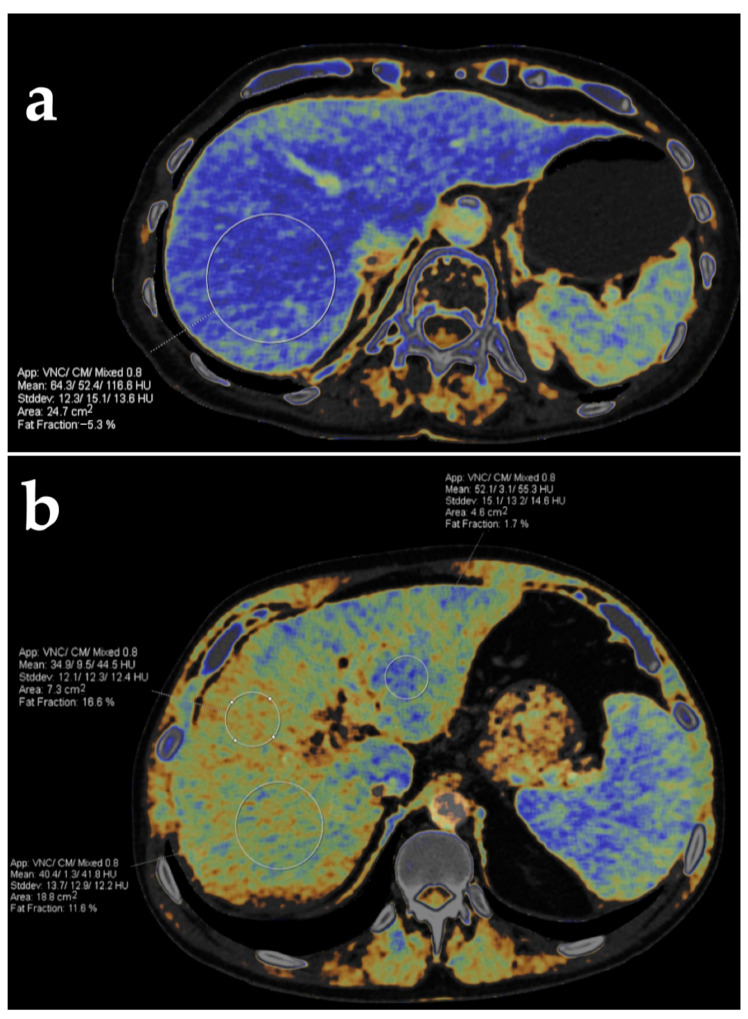
Liver fat quantification using dual-energy CT in two different patients. Fat fraction color-coded maps generated from DECT images in the axial plane show no fat infiltration in the first patient (**a**) and different fat fractions obtained from three regions of interest in another patient (**b**).

Dual-energy CT can provide reliable material composition estimation without the need for unenhanced acquisitions (Figure 15) [6,69,70]. However, it has shown lower performance for fat quantification compared to SECT and has a moderate correlation with MR spectroscopy [66]. Still, a significant correlation has been found between liver fat percentage obtained from a multi-material decomposition algorithm and attenuation measurements. A 10% threshold of liver fat was highly sensitive and specific for predicting the non-contrast CT attenuation indicative of moderate to severe steatosis [65]. Others found a strong correlation between hepatic fat fractions obtained from DECT and those obtained from MRI, without observing significant differences across different scanning phases [71].

For VNC images, a liver attenuation of <40 Hounsflied units (HU) is highly specific and positively predictive for moderate to severe steatosis using unenhanced SECT criteria, supporting their reliability despite the mild overestimation of liver attenuation compared to true unenhanced images (by 5.4 UH) [72]. A recent study performed using proton density fat fraction MRI as the reference standard found only a moderate correlation between VNC attenuation values and liver fat content with 57–68% sensitivity, but discovered high (≥90%) specificity for diagnosis of steatosis [73]. Others found comparable diagnostic performance of CT parameters measured in VNC and true unenhanced images for diagnosing liver steatosis [74,75,76]; however, further studies are needed if we are to be able rely on VNC values for the diagnosis of liver steatosis.

On the opposite side, hepatic fat quantification of low-dose unenhanced DECT (volumetric CT dose index of 2.94 mGy) strongly correlates with MRI proton density fat fraction and provides an excellent diagnostic performance for diagnosis of the fatty liver with a cutoff value of ≥4.61%. This method could be an option for specific workup of hepatic steatosis [77].

Early studies have also used attenuation differences and curves from low- and high-energy VMIs to grade the severity of fat infiltration. Given its unique property of greater attenuation of high-energy X-rays compared to low-energy X-rays, fat would appear brighter on subtracted images (lower-keV image subtracted from higher-keV image) [67].

Iodine concentration values during the portal venous phase may additionally improve the diagnosis of steatosis in contrast-enhanced CT, being significantly lower in fatty livers compared to healthy liver patients, which is possibly due to a lower interstitial distribution of iodine [78].

##### Iron Deposition

Iron deposition occurs in chronic liver diseases of multiple causes. This may lead to liver damage and increases the risk of cirrhosis and HCC [6,8,12,79].

The difference between liver attenuation at high- and low-energy acquisitions has shown strong linear correlation with MR R2* relaxometry techniques for estimating iron deposition [6,80]. Virtual iron content determination (3-material decomposition algorithm) has also shown a correlation with serum ferritin levels and displayed sensitivity and specificity comparable to MR R2* relaxometry, but only for clinically relevant liver iron concentration thresholds [6,81]. Despite the good diagnostic accuracy for cases of moderate to severe iron overload, DECT quantification results vary where MRI quantification is limited due to extremely rapid signal decay [8,79], especially for low-grade deposition [8], and a large cohort is still needed for validation to confirm the accuracy and robustness of the method [6,24,82]. Furthermore, maximized spectral separation is important and patient size may impact iron quantification [83].

The opposite effect of fat and iron deposition in CT attenuation limits the assessment of their concurrent accumulation on SECT. Being capable of specific material quantification, DECT may overcome such limitation [8]. However, significant fat deposition may lead to an underestimation of iron accumulation [84]. Being based on a three-tissue decomposition algorithm (iron, soft tissue, and fat), virtual iron content images has the potential to eliminate the effect of fat on iron quantification, but this still requires further investigation [80,85].

##### Fibrosis

The staging of liver fibrosis is relevant for patient prognosis as early stages may be reversed by treating or controlling the causative factor. The most commonly used imaging tests for diagnosis are MRI and ultrasound elastography. Several CT methods for staging fibrosis have been described but may require specialized software and high radiation dose [8].

The extracellular space expansion induced by the deposition of collagen fibers is strongly correlated with the degree of fibrosis and may be quantified [34]. The hepatic extracellular volume fraction (fECV) on SECT, indicating the absolute contrast enhancement at the equilibrium phase, requires two acquisitions, larger amounts of contrast media, and is prone to misregistration errors. Dual-energy CT allows researchers to perform an accurate quantification of iodine contrast material based only on the equilibrium phase. Multiple studies have shown good results in estimating the degree of fibrosis [64,86]. Both equilibrium phase images at 180 s and 240 s have been used (the latter with slightly greater correlation coefficient) [64]. A 10 min phase has also been proposed, theoretically allowing for greater diffusion of contrast [87]. However, a different study obtained similar results with equilibrium phase images at 180 s and 10 min [88]. Still, existent studies are heterogeneous, with different scanners and protocols, and many rely on liver biopsy specimens, predisposing these methods to staging misclassifications due to heterogeneous fibrosis distribution [64]. Therefore, validation of the use of fECV calculation in DECT for liver fibrosis quantification requires further investigation [8,24].

**Figure 16:**
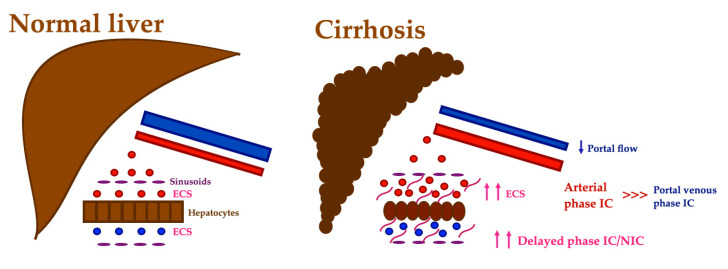
Liver fibrosis staging with dual-energy CT. The extracellular space (ECS) expansion induced by fibrosis may be quantified by measuring iodine concentration (IC), which increases in the delayed phase. Additionally, reduced portal flow with hepatic arterial buffer response in liver fibrosis leads to reduced IC during portal venous phase and increased IC during the arterial phase. NIC—normalized IC.

The hepatic fECV has also been obtained from iodine density maps [89]: liver fibrosis leads to reduced portal flow, with a consequent drop in IC, and the hepatic arterial buffer response increases the IC during the arterial phase (Figure 16) [90]. Several studies have shown good correlation between IC and NIC and histologic fibrosis and cirrhosis, although different scanning phases were used [89,90,91,92]. The ratio between IC at arterial and portal venous phases is another promising quantitative parameter [90,93]. Iodine slopes calculated from liver IC at the equilibrium phase, as well as either the arterial or portal venous phase, also correlate positively with the model for end-stage liver disease (MELD) score [94].

A lower iodine washout rate, calculated from portal venous and 3 min delayed phases, has also been reported in patients with clinically significant fibrosis and cirrhosis, providing improved accuracy compared to fECV [95].

#### 3.2.4. Trauma

The liver is the second most commonly injured abdominal organ in polytraumatic patients. Important treatment decisions are often performed based on initial CT scans in order to stratify patients who need immediate surgical or angiographic management. Dual-energy CT methods can provide valuable information about the visceral enhancement, vascular injury, and presence of active bleeding. They can accurately differentiate between hemorrhage and calcification and reduce metal-related artifacts that may lead to serious interpretation difficulties [96]. Low-keV images improve the detection of organ lacerations [96,97].

#### 3.2.5. Vascular Applications

The use of low-keV VMIs allowed us to obtain better objective and subjective image quality for the evaluation of hepatic vasculature [98,99,100]. These allowed for a 25.4% reduction in iodine contrast load [101].

**Figure 17:**
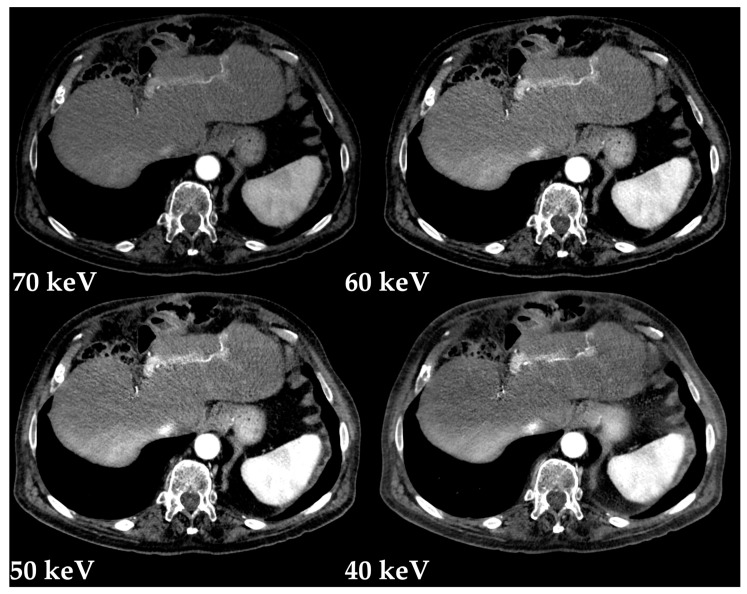
Improved vascular depiction with low-energy VMIs (Monoenergetic Plus advanced noise-optimized algorithm) in a scan with poor intrahepatic contrast enhancement due to the inadequate timing of acquisition.

Several factors may impair the quality of liver enhancement, including chronic liver disease, altered hemodynamics, inadequate timing of acquisition, and contrast dose savings in patients with renal dysfunction. Low-energy VMIs showed significant CNR improvement in images obtained with poor intrahepatic contrast enhancement (Figure 17) [102].

Iodine concentration in the portal venous phase is a promising noninvasive measure for accurately assessing portal venous hypertension, showing strong correlation with direct portal venous pressure [103]. Furthermore, it can be used to assess liver blood flow changes after transjugular intrahepatic portosystemic shunt placement based on changes in the quantitative indices of iodine density [104].

Up to 44% of patients with HCC develop malignant portal venous thrombosis (PVT), worsening prognosis and limiting treatment options. Therefore, it is important to distinguish this from bland thrombosis. Thrombus enhancement in the late arterial phase is the most specific sign which suggests a malignant nature. Iodine maps obtained from DECT allow for an accurate noninvasive characterization of PVT, although the optimal scanning phase for thrombus quantification and the optimal iodine threshold are yet to be defined [6,105]. The use of low-keV VMIs (best at 40 keV) significantly improved the diagnostic performance for the detection of PVT compared to other DECT reconstruction algorithms [106].

### 3.3. Limitations

Current DECT technology is still limited by the significant inter-vendor variability in material-specific decomposition methods, such as in terms of monochromatic data and iodine quantification. Attenuation measurements in VNC images in different organs and scanner settings also need to be compared among those of other vendors. Besides, iodine quantification accuracy is also influenced by other factors related to the patient (e.g., body habitus size) and scanner generation [6].

Dual-energy CT is prone to unique artifacts related with the image post-processing, including subtraction-related artefacts (e.g., inadvertent subtraction of calcifications) [107]. Small lesions are susceptible to volume-averaging originating pseudoenhancement and treated avascular lesions may resemble simple cysts [5].

Workflow limitations related to the increased reconstruction time have been improved with multiple strategies, including the use of lighter workstations or integration of postprocessing software in remote workstations [6]. However, large amounts of data require capacious storage systems and demand more interpretation time [73].

Clinical practice decisions, including patient and exam type selection for dual-energy scanning and image reconstruction, have also been limitative. A multi-institutional consensus suggested the use of standardized abdominopelvic CT protocols, recommending DECT in specific scenarios [108].

### 3.4. Future Perspectives

Radiomics analysis uses advanced mathematical algorithms to extract information from imaging data in order to generate patterns of pathological processes that are beyond visual image interpretation. Although limited, promising results have been published for the potential applications of DECT-based radiomics, including in areas of nodal metastasis prediction in several cancers, the differentiation of solid benign and malignant liver and pancreatic lesions, or the discrimination of normal liver from steatosis and cirrhosis [6,109,110,111]. However, the limited reproducibility of radiomic features from DECT has been reported [112].

Deep learning imaging reconstruction techniques performed using convolutional neural networks improve CT image quality [8]. These algorithms have demonstrated improved conspicuity of hepatic lesions on DECT [113,114,115], and the automatic localization and classification of liver lesions [116]. Artificial intelligence is also capable of improving liver segmentation and fat quantification as well as predicting the occurrence of metastatic disease [117,118].

The capability of differentiating between contrast agents with different attenuation properties at low- and high-energy has allowed researchers to perform simultaneous imaging with two contrast agents, providing different vascular phases in a single acquisition [119].

Novel photon-counting detectors convert incident X-rays into individual moving charges that create a current and a signal directly proportional to individual photon energy [6]. Providing more discrete information, photon-counting CT imaging enables K-edge imaging and the differentiation of more than two materials, [4] improving the conspicuity and delineation of tumors. Thinner slices at equivalent radiation doses reduce partial volume averaging. Furthermore, the occurrence common deleterious image artifacts is markedly reduced with the elimination of electronic noise and dose efficiency is improved [120]. An in silico study provided dual-contrast liver imaging with a single-scan after the sequential injection of a gadolinium-based contrast agent (providing arterial enhancement) and an iodine-based contrast agent (providing portal venous enhancement) [121]. This was also validated in rabbits. Future studies are needed to determine the optimal parameters for a clinical protocol [122]. The liposomes or nanoparticles needed to incorporate less biocompatible molecules are under investigation [123].

## 4. Conclusions

Dual-energy CT has unique capabilities of material differentiation and quantification, provides improved diagnostic performance among many disease processes, allows for the reduction of iodine contrast and radiation dose, among many other advantages over the SECT method. Its value has been proven in many clinical applications, including in the treatment of liver disease. Lesion detection and characterization, accurate staging and treatment response assessment, non-invasive quantification of fat/iron liver deposition and fibrosis, and thrombi characterization are among the most valuable uses of DECT (Table 1). Current limitations include reduced image quality with larger body sizes, cross-vendor and scanner variability, and long reconstruction time. Deep learning imaging reconstruction and the novel spectral photon-counting CT methods are promising techniques for obtaining improved image quality and diagnostic accuracy with lower radiation dose.

## Figures and Tables

**Table 1:** Summary of current applications of dual-energy CT in liver pathology.

Pathology	Application [Reference Number]
Lesion detection and characterization	- Low-energy VMIs (40–55 keV) improve the detection of hypervascular [2,16,17,19,20,21,22,23] and hypovascular liver lesions [34,35,36,37]. - Spectral attenuation curves allow for discrimination between benign and malignant liver lesions [3,25,26]. - Iodine quantification in HCC can predict microvascular invasion [28,29,30,31,32] and early recurrence after resection [33]. - Iodine quantification [41,42,43] and spectral attenuation curves [5] are helpful in characterizing small hypoattenuating liver lesions. - Quantitative analysis may help to differentiate liver metastasis from abscesses [44], necrotic HCC [45] or small intrahepatic mass-forming cholangiocarcinomas [46].
Treatment response evaluation	- Iodine quantification may be a reliable biomarker for tumor viability in HCC treated with antiangiogenic drugs [5,48], radiofrequency ablation [49], microwave ablation [51,52], TACE [53,54,55,57,58], and yttrium-90 radioembolization [60]. - Iodine tumor burden criteria for posttreatment evaluation in liver metastasis from GIST outperforms RECIST 1.1 and mChoi criteria [63].
Diffuse liver diseases	- Liver fat percentage obtained from a multi-material decomposition algorithm shows a strong correlation with fat fractions obtained from MRI [71]. - Virtual iron content determination (3-material decomposition algorithm) shows comparable sensitivity and specificity to MR R2* relaxometry, but only for clinically relevant liver iron concentration [6,82]. - The hepatic extracellular volume fraction (fECV) obtained with DECT, either from the absolute contrast enhancement at the equilibrium phase [64,86] or from iodine quantification, shows good correlation with liver fibrosis and cirrhosis [89,90,91,92].
Trauma	- Low-keV images improve the detection of liver lacerations [96,97]. - Iodine-selective images are useful for evaluating visceral enhancement, vascular injury, and the presence of active bleeding [96,97].
Vascular applications	- Low-keV VMIs improve the evaluation of hepatic vasculature [98,99,100,102] and allow for a 25.4% reduction in iodine contrast load [101]. - Iodine concentration is a promising tool for assessing portal venous hypertension [103] and blood flow changes after TIPS placement [104]. - Low-keV VMIs (40 keV) improve the detection of PVT [106] and iodine maps allow for differentiation between malignant and bland PVT [6,105,106].

CT, computed tomography; DECT, dual-energy computed tomography; fECV, extracellular volume fraction; GIST, gastrointestinal stromal tumor; HCC, hepatocellular carcinoma; keV, kiloelectronvolt; MRI, magnetic resonance imaging; PVT, portal venous thrombosis; RECIST, Response Evaluation Criteria in Solid Tumors; TACE, Transarterial chemoembolization; TIPS, transjugular intrahepatic portosystemic shunt; VMIs, virtual monochromatic images.

## Data Availability

Not applicable.

## Abbreviations

CNR, contrast-to-noise ratio; CT, computed tomography; DECT, dual-energy computed tomography; fECV, extracellular volume fraction; FOV, field of view; GIST, gastrointestinal stromal tumor; HCC, hepatocellular carcinoma; HU, Hounsflied units; IC, iodine concentration; keV, kiloelectronvolt; kVp, kilovolt peak; MELD, Model For End-Stage Liver Disease; MRI, magnetic resonance imaging; NAFLD, non-alcoholic fatty liver disease; NIC, normalized iodine concentration; PVT, portal venous thrombosis; RECIST, Response Evaluation Criteria in Solid Tumors; RFA, radiofrequency ablation; SECT, single-energy computed tomography; TACE, Transarterial chemoembolization; TIPS, transjugular intrahepatic portosystemic shunt; VMIs, virtual monochromatic images; VNC, virtual non-contrast.
